# A Community of Practice to increase education and collaboration in dementia and ageing research and care: The Liverpool Dementia & Ageing Research Forum

**DOI:** 10.1111/hex.13806

**Published:** 2023-06-26

**Authors:** Clarissa Giebel, Hilary Tetlow, Thomas Faulkner, Ruth Eley

**Affiliations:** ^1^ Department of Primary Care and Mental Health University of Liverpool Liverpool UK; ^2^ NIHR Applied Research Collaboration North West Coast Liverpool UK; ^3^ SURF Liverpool Liverpool UK; ^4^ Mersey Care NHS Trust Sefton UK; ^5^ Together in Dementia Everyday (TIDE) Liverpool UK

**Keywords:** carers, Community of Practice, dementia, public and patient involvement and engagement

## Abstract

**Background:**

Too often, dementia research is conducted in research silos without thorough integration and the involvement of people with lived experiences, care professionals and the Third Sector. Research can also get lost in academic publications, without reaching those benefiting most from the evidence. The aim of this methods and evaluation paper was to outline the aims, components and evaluation of the public‐facing and ‐engaging Liverpool Dementia & Ageing Research Forum, to provide a blueprint for setting up similar communities of practice.

**Methods:**

The Forum was set up in 2019 with the aim to (a) connect different stakeholders in dementia and ageing and co‐produce research and to (b) inform and educate. This paper provides an account of the Forum model and evaluates the following key elements: (1) engagement; (2) experiences of the Forum and its impact (via an online evaluation survey and three reflections). All Forum members and attendees were asked to complete a brief evaluation survey about their experiences from October to November 2022. Three regular Forum attendees provided a case study about their involvement and its impact.

**Findings:**

The Forum has reached out to diverse stakeholders and the general public, generating growing interest and engagement since its initiation. Forty‐four members and attendees completed the survey. Most attendees completing the evaluation survey have so far engaged in between 5 and 20 activities (47.8%), and 91% felt the aims of the Forum have been met. Engaging in the Forum has produced various benefits for attendees, including increased research capacity and knowledge, as well as improved connectivity with other stakeholders. Eleven percent of respondents, 39% of lived experts, stated they experienced improved access to postdiagnostic care.

**Conclusions:**

This is the first reported multistakeholder Community of Practice (CoP) on dementia and ageing. We make key recommendations for setting up and running similar dementia CoP, as they provide a noninterventional format for raising awareness, capacity and access to dementia care.

**Patient and Public Involvement:**

This paper reports on the involvement and engagement of people with dementia, unpaid carers, health and social care providers and Third Sector organisations in a CoP.

## 1 INTRODUCTION

Patient and public involvement and engagement (PPIE) in dementia research are increasingly reported.^1^, ^2^ PPIE is critical to engaging people with diverse nonacademic work, voluntary and lived experiences in the field of research. To ensure that the voices of nonacademic team members in the design, conduct and dissemination stages are truly heard and recognised, stakeholders need to be integrated equitably in any research—this includes providing support to conduct some qualitative analysis and code transcripts, co‐design topic guides and surveys and be co‐authors in publications and nonacademic outputs, such as lay summaries.^3^, ^4^ With some research involving specifically recruited individual public advisers,^5^, ^6^, ^7^ other research engages with dementia and carer stakeholder groups such as the Dementia Engagement and Empowerment Project^8^ and the European Working Group of People With Dementia.^9^, ^10^

A broader approach to involving lived and professional experts in the co‐design of research, and also empowering and advancing their knowledge, is by creating a Community of Practice (CoP). Communities of Practice can help facilitate wider and more in‐depth social learning and knowledge exchange compared to individual learning.^11^ Specifically, communities of practice bring together peers and professionals and experts from similar backgrounds, such as dementia, and enable learning through social engagement and joint participation in social practices, such as attending topic‐specific group events and activities and engaging in topical discourse and discussion. Developed by Wenger,^12^, ^13^, ^14^ this social theory has been employed in various settings outside of the educational sector, and in dementia, for example, has been found to effectively bring together a diagnostic clinical network^15^ and primary care memory clinics.^16^ However, it appears that no CoP, to date and reported in the literature, has been brought together to share knowledge about dementia care and to jointly generate research ideas for collaborative delivery, with diverse stakeholders from academia, health and social care, the voluntary and community sector and lived experience.

The Liverpool Dementia & Ageing Research Forum was set up in September 2019 with two underpinning aims: (1) to educate and inform about the latest dementia research and services to anyone with a professional or personal interest in the topic; and (2) to build an interdisciplinary wide network of stakeholders from academic, care provider, policy, Third Sector (voluntary services/charities) and lived experiences backgrounds to co‐produce research together. The Forum provides a range of activities to network and collaborate across different stakeholder groups, generate research ideas, jointly apply for research funding and inform and connect members about the latest research, care services and related opportunities.

The aim of this methods and evaluation paper was to outline the aims, components, and evaluation of the public‐facing and ‐engaging Liverpool Dementia & Ageing Research Forum, to provide a blueprint for setting up similar Communities of Practice. For this purpose, we have conducted a mixed‐methods evaluation of the Forum, involving an online survey to all previous attendees and ongoing members, as well as three case studies on different stakeholders who have been involved in the Forum (one carer, one service provider, one clinician turned academic).

## 2 METHODS

### 2.1 The Liverpool Dementia & Ageing Research Forum

The international Forum, which is based in Liverpool, comprises four regular activities, which address each or both aims of the Forum to different degrees, enabling as large an engagement and skill‐building of attendees and members as possible: (1) bimonthly public seminars/webinars; (2) monthly journal clubs; (3) biannual regional networking meetings and (4) an annual conference. Public seminars turned into webinars during the COVID‐19 pandemic and have remained so. Seminars are free to the public and open to anyone with an interest in dementia and ageing. At each webinar, a different speaker shares their research or overview of care provision, which is then discussed with the audience via a moderator. Previous topics included carer resilience, dementia in South Asian minority groups, innovative long‐term care for dementia, falls in older adults, Dementia Care Navigation and Admiral Nurses, as well as social and spatial inequalities in healthcare use for people living with dementia.

Monthly journal clubs are open to members interested in discussing the latest dementia research in the field and are normally attended by postgraduate students, academics, clinicians and Third Sector providers. To date, these have been held remotely to enable people outside the University of Liverpool to join easily.

Regional networking meetings are taking place twice a year, virtually to date, and last up to 90 min. Each meeting involves two regional speakers presenting their care services or initiatives, followed by a discussion and a virtual roundtable update and discussion on ongoing services, initiatives and research. This provides a more open platform for networking and collaboration.

The annual conference originally started face‐to‐face and has returned to face‐to‐face delivery after 2 years of remote delivery. Based on the continued growth of membership and attendees, the fourth annual conference was the first to invite open abstract submissions from academics, students, care providers and people with lived experiences of dementia and ageing.

#### 2.1.1 How is it maintained?

The Forum was set up by C. G., as a result of recognising diverse expertise in the dementia care field across the region, despite a lack of a coherent network or Forum to engage with and learn from one another. The Forum is thus led and organised by C. G., with additional logistical and planning support provided for the annual conference and regional networking meetings (abstract submissions, venues and room bookings and refreshment orders). The Forum is advertised via email and word of mouth to the National Health Service (NHS) Trusts, Third Sector organisations, unpaid carers, as well as via social media, the University of Liverpool and National Institute for Health and Care Research Applied Research Collaboration North West Coast (NIHR ARC NWC) news, Eventbrite and its own webpage.

Forum members receive a monthly newsletter about event information and research and engagement opportunities, and anyone can join as a Forum member. An accompanying webpage provides additional news updates, and people can register for events via Eventbrite.

#### 2.1.2 Data collection

Four different sources of data were collected: (1) registration and attendance numbers; (2) co‐produced research and publications; (3) evaluation survey and (4) three case studies on engagement, experiences and impact.

Registration and attendance numbers were taken from Eventbrite and Microsoft teams/Zoom/in‐person events. Data on funded grant applications and publications were recorded continuously on an Excel spreadsheet. The evaluation survey was designed by the Forum organiser (C. G.) and piloted with a senior healthcare professional and academic. The survey was set up on google forms, and all survey questions are listed in Table A1. Three purposefully varied stakeholders and lived experts were approached about their engagement, experiences of involvement and the impact this has had on themselves as a person and within their professional/caring role.

No ethics approval was required as the feedback and evaluation survey on engagement and experiences of the activities of the Forum involved registered Forum members, and no participants were recruited for this feedback.

#### 2.1.3 Data analysis for evaluation

Data (survey responses) were analysed by google forms automatically, generating frequencies of responses for each question, graphs and diagrams.

#### 2.1.4 Recommendations for similar CoPs

Based on the host's experiences of running the Forum, and on the member's experiences of engaging with the Forum, this paper provides key recommendations for setting up and running a similar CoP.

## 3 RESULTS

### 3.1 The Forum in numbers

#### 3.1.1 Participation and engagement

**Figure 1:**
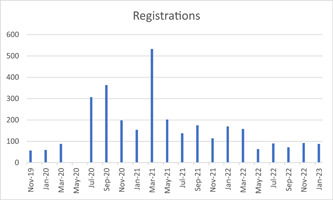
Registrations for bimonthly seminars/webinars. The first three seminars were held face‐to‐face, and due to COVID‐19 the May seminar was moved and formed into a webinar in July. All subsequent talks have been held in webinar format and will continue to do so.

Registration numbers for the bimonthly seminar/webinar series are shown in Figure 1. The first three seminars took place face‐to‐face, with the format moved online due to COVID‐19 after the March webinar series. As shown, this enabled a large increase in people registering for the webinar series, resulting in increased attendance numbers. The greatest level of interest was reported for a webinar on a dementia core outcome set (*n* = 533),^17^ the impact of COVID‐19 on dementia care (*n* = 364)^6^, ^18^ and caring for a parent with dementia from a distance (*n* = 307).

#### 3.1.2 Co‐produced research and publications

The Forum has generated a number of different co‐produced research ideas, with 10 funded projects focusing on the impact of COVID‐19 on dementia social care in the community, the impact of COVID‐19 on care home visitation experiences and the care home workforce, global research into dementia care with Colombia, India and Uganda, as well as the unmet mental health needs of paid and unpaid carers, inequalities in accessing and using dementia care and inequalities in social care needs assessments. These were, to date, funded by the NIHR, the University of Liverpool ODA Seedfund and the Policy Support Fund, The Pandemic Institute, Geoffrey and Pauline Martin Trust.

We are also having NIHR ARC NWC funded PhD students with topics generated as a result of the Forum connections, as well as NIHR ARC NWC research interns who are health and social care practitioners, conducting linked Forum research into dementia. Examples of internship projects include the experience of driving assessments in dementia and evaluating the impact of singing and dancing groups for people living with dementia and their carers.

To date, co‐produced Forum research has resulted in 23 publications and policy briefings.^18^, ^19^

#### 3.1.3 Embedding within existing infrastructure

The Forum emerged from the NIHR ARC NWC which involves 60 diverse member organisations. The Forum drew upon the ethos of the equitable and collaborative ARC to set up a dementia and ageing‐specific network of lived and professional experts. While the Forum started in the North West Coast region, it grew to become a national and international Forum, facilitated by remote webinars and events. When required, the ARC NWC provides minimal administrative support for hosting the annual conference and room bookings for face‐to‐face networking meetings, and support with public adviser fee payments for those lived experts who become actively involved in specific grant applications.

### 3.2 Evaluation survey

#### 3.2.1 Demographics

Forty‐four Forum members and users completed the evaluation survey between October and November 2022. Survey responders comprised a mix of academics, health and social care professionals, postgraduate students and lived experts, and from a mix of within the Liverpool region (*n* = 24, 54.5%), UK‐residing outside Liverpool (n = 14; 31.8%) and from outside the United Kingdom (*n* = 6; 13.7%).

#### 3.2.2 Engagement with activities

**Figure 2:**
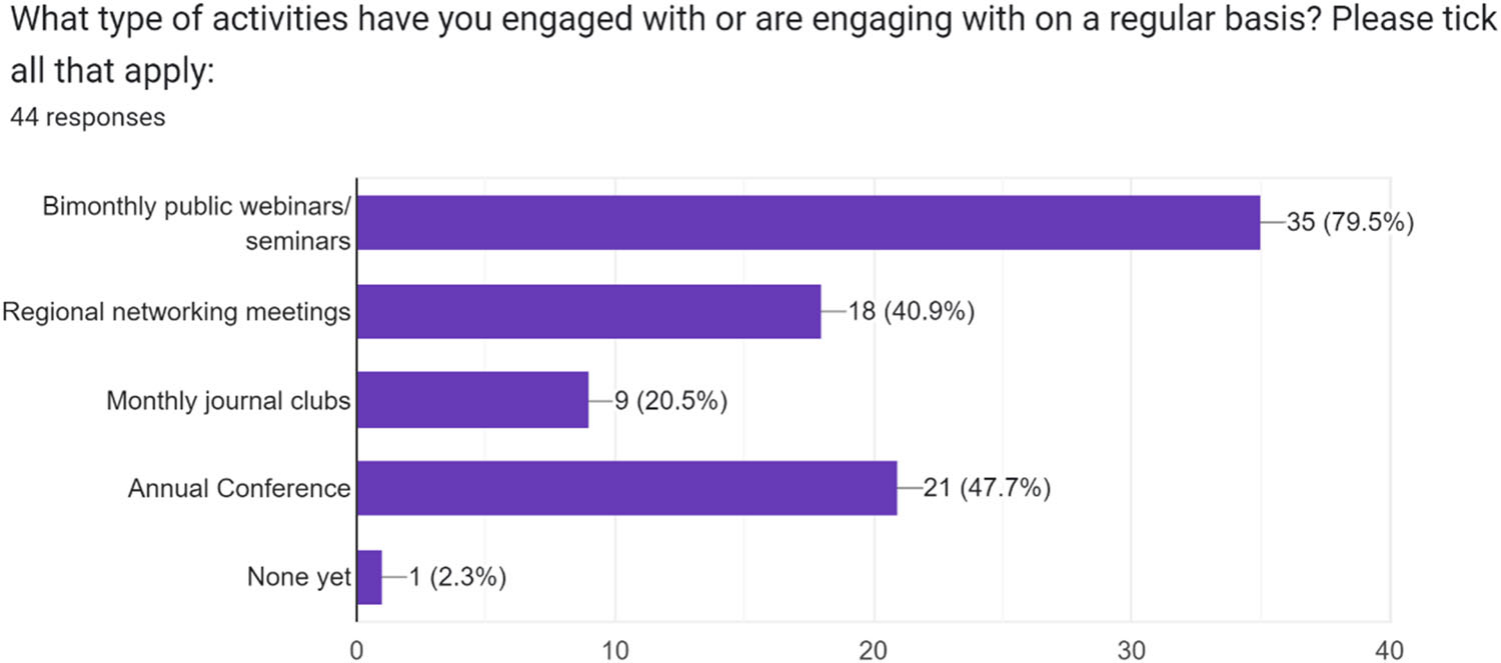
Engagement with different forum activities.

Engagement with activities ranged from ‘0’ to date to over 20, with the majority of respondents having participated in between 1–5 (45.5%) and 5–10 (34.1%) activities. The most commonly engaged activity of all four was bimonthly public webinars/seminars (*n* = 35; 79.5%), with one respondent not having participated in an activity yet as a newly added member (see Figure 2).

#### 3.2.3 Feedback

The vast majority of responders stated that the aims of the Forum were met (91%), with 7% stating ‘maybe’ and one person stating ‘no’. When asked why responders were attending Forum events, 88% stated ‘to find out more about the latest evidence research and evidence’, with 64% stating ‘to meet peers and connect with different stakeholders and experts in the field. Over a third wanted ‘to jointly develop and apply for research funding and get involved in research’. As a result of the Forum, two‐thirds of responders have met experts in the field and learned about different services, with nearly half of the responders having engaged in research by co‐producing research. Eleven percent (*n* = 5) also noted improved access to support services. Of all lived experts who shared their views (*n* = 13), this equates to 38.5% who reported positive impacts on access to care.

All responders had positive to very positive (93%), or neither negative nor positive experiences of the Forum.

### 3.3 Three member reflections

#### 3.3.1 Reflection 1—Unpaid carer and support service provider

##### Background

As a former Consultant in Fashion Merchandising, I gave up my company to look after my mother who had developed Alzheimer's and she died peacefully at home. I also looked after her brother who had vascular dementia and their half‐sister who had vascular dementia. They all had long lives between their late 80s and 90s when they died, and it is from them that I get my belief in giving back to the community.

I am active in Merseyside and beyond in the following, to name some of my key involvement roles: Deputy Lead governor of Mersey Care NHS Foundation Trust and have been a governor since it became a Foundation Trust; a founding member, co‐chair and treasurer of SURF (Service Users Reference Forum) for people and carers of people with dementia working together to try to make changes in attitudes and services; on the Liverpool Dementia Action Alliance co‐ordinating group; involved in the patient engagement and experience group of Liverpool Place; Founder member of Together in Dementia Everyday (TIDE) carers involvement network supported by The National Lottery; Chair of the Liverpool Experts by Experience of the Doctorate in Clinical Psychology and a Public Advisor for the NIHR Applied Research Collaboration North West Coast.

I have spoken at conferences and helped run conferences. Set up and run groups for people with dementia and carers, I will take part in anything where I can promote awareness of dementia and the importance of listening to experts by experience.

##### My engagement and experiences with the forum

I have worked tirelessly with C. G. [Forum organiser] on research projects, especially during Covid‐19 as it had disastrous results on both carers and people with dementia, and with all my contacts with carers and people with dementia, I know what is happening in the community. From the start of any research project when it had almost nothing more than a title, C. G. would ask if I wanted to be involved as a public advisor and as a carer then at the first meetings with the full research group, we hone down the project and apply for funding. If funding is granted through the information gathering, we check what information is coming through against what I and usually another carer are hearing from our grassroots information this is especially important as it acts as a check. This is the area where my knowledge is invaluable to the research project.

##### Impact

Being involved with the Forum and research has enabled me to understand my worth and not be backward in coming forward that what I must contribute is as important and valid as the professionals in the research and specialist field. I get much out of the Forum as I get to hear of research that I had no idea was/is happening. It also has provided me with the speaker to come to SURF and talk to our group that can run to 30 people, but our notes go to over 100. The skills I have developed during this time really are to do with the knowledge I have developed plus not to sit quietly in discussions but to take part as I have as much knowledge as any of the experts.

#### 3.3.2 Reflection 2—Charity representative

As the Chair of the Board of Trustees for TIDE—I want to keep in touch with emerging research in the dementia world to inform our campaigning and influencing activities. We believe that carers and former carers have the experience and knowledge to improve health and social care, research and policy development throughout the UK.

A social worker by profession, my background is in health and social care, having worked in local government, the Department of Health in England and as an independent consultant. I have been a Board member for Wales and West Housing Association for the last 8 years and have also previously chaired the Parkhaven Trust, a Merseyside charity providing residential and day services for older people and people with dementia.

I have a strong interest in research and represent TIDE in several research advisory groups, recruiting and mentoring unpaid carers to participate as experts by experience, to ensure their voices are heard in shaping and monitoring programmes. Membership of the Forum has led to TIDE's participation in several research programmes and I have co‐authored subsequent publications.

It can be hard to keep up to date with wider research findings, so the Forum provides a great opportunity to find out about current work. Also important is the opportunity to network with colleagues from the research world. Ensuring that research findings are disseminated and applied is a constant challenge and the Forum plays an important role in this regard. Recent research by Forum members into the impact of COVID on older people, people with dementia and unpaid carers has provided important evidence for TIDE in our campaigning work. Membership in the Forum has given me greater insight into the research world as well as enhanced my knowledge about ethics, methodology and data analysis.

The recent annual Forum conference held in Liverpool gave TIDE the opportunity to promote the importance of involving unpaid carers in research and some practical examples of projects we have undertaken that involve carers as equal partners.

#### 3.3.3 Reflection 3—Trainee clinician turned researcher

##### Background

I am based in Southport and have worked in mental health for around 14 years. This experience has covered acquired brain injury and adult mental health, and in my current role of 7 years as an assistant psychologist in older adult mental health. It was through my current role that I began supporting families affected by dementia. My role has involved delivering individual and group support around postdiagnostic support, carers and mild cognitive impairment.

Through working as an assistant psychologist, I regularly heard in‐depth accounts from people on their experiences of living with dementia and adjusting to a diagnosis. I have also developed an appreciation for the huge challenges faced by people and families living with dementia, as well as an understanding of the limitations of the health service in providing quality care, particularly for people with young‐onset dementia.

Over time, I began to request opportunities to develop research or evaluations on where services could improve for people using them. Unfortunately, time and resources are often limited when working in the NHS, and it was often difficult to conduct research.

### 3.4 Experiences with the Forum

I initially got involved with the Forum through a research internship with the National Institute of Health Research, which was led by C. G. For this I produced a qualitative project which is now being processed for publication. The completion of this internship has given me the opportunity to lead a research project including the designing, analysis and writing up. I have also had the opportunity to present at an international conference and display a poster at the Research and Forum conference in Liverpool.

Every month I attended a virtual meeting called a Journal Club in which a mixture of academics, clinical workers and other researchers discuss research that is presented by an attendee. I have found these meetings particularly helpful, and it has been interesting to listen to interpretations from a variety of perspectives. The meetings are less than an hour long and the virtual aspect ensures they are highly accessible. I feel these meetings are highly valuable for someone like myself who is looking to increase my academic competencies while working in a clinical setting. I feel that my attendance at these meetings has increased my ability and confidence to critically review research articles, as well as the educational aspect from the content of the studies.

Finally, the Research Forum is hugely valuable for community networking. There are regular webinars and virtual events, as well as an annual conference. These have allowed me easy access to recent research, as well as making it very easy to develop connections with local and national charities. Community support can often be dynamic, experiencing lots of change regarding locations and personnel and it is important to be able to keep up with its new initiatives and research. I have found the research forum to be a vital method of dementia networking in the North West as I see the positive difference in community inclusion that is present in Liverpool, but not in the area I currently work in.

### 3.5 Key recommendations for setting up and running a dementia CoP

Based on the experiences of the Liverpool Dementia & Ageing Research Forum, and challenges encountered (primarily the COVID‐19 pandemic restricting face‐to‐face meetings, and at times staff time issues), we have five key recommendations for setting up and running a dementia CoP:

1. *Have a supportive infrastructure to launch the CoP from*: In the case of our Forum, this included the NIHR ARC NWC. When setting up a new dementia CoP, it is beneficial to link in with existing supportive academic, health or social care infrastructures, as it will also enable growing the CoP.
2. *Involve members to help run different activities*: While all activities are organised by one Forum lead, larger events such as the annual conference or the regional networking meetings require additional support in terms of content and logistics planning. By involving local members from diverse backgrounds to support the planning of the annual conference or sourcing refreshments and rooms for the regional networking meetings, the Forum can draw on the existing expertise of its members and attendees and also allow wider buy‐in and attendance at Forum events.
3. *Engage with people via different complementary activities and communication streams and platforms on a regular basis*: In the present case, this includes providing four regular different activities and events, as well as monthly email updates, and newsletters and engagement via social media platforms. These activities emerged from growing discussions and facilitation opportunities within the Forum.
4. *Enable equitable access and ensure everyone can contribute and get heard*: As part of the Forum, we ensure that people with lived experiences as well as professionals, and academics and students can attend and share their views and contribute to the Forum (including via providing talks, shaping research ideas or leading research). This also involves a flexible approach by offering both face‐to‐face and remote events, as not all attendees may wish to attend face‐to‐face (especially when living in other regions of the country or other countries altogether) or to attend remotely (due to digital difficulties). This flexible approach particularly emerged from the COVID‐19 pandemic and related face‐to‐face restrictions.
5. *Evaluate the CoP and ask what works and what may not work so well, to increase engagement and feelings of building the CoP together*: The brief anonymised online evaluation of the Forum as well as the three reflections enabled members to express their views about the Forum. This was the first thorough evaluation of the Forum, with ongoing verbal feedback provided by attendees and members at different Forum events. For example, the fact that regional networking meetings are now taking place face‐to‐face again was based on Forum feedback at the event of the last regional networking meeting.

## 4 DISCUSSION

This paper provides a methodological overview of setting up and running a public‐facing, inclusive and collaborative research and engagement forum into dementia and ageing. Since initiation, the Forum has engaged with diverse stakeholders in the field and members of the general public, bringing together academic and care professionals, students, decision‐makers and lived experts in the field to jointly shape and develop research and share new evidence into care, thus successfully meeting its aims.

As a result of engaging with the Forum, this CoP has led to diverse research involvement and co‐production by involving various nonacademic stakeholders in grant submissions and thus research projects. Stakeholders and experts without previous connections were brought together based on their shared lived and professional expertise in dementia and ageing, evidencing how a CoP can generate new connections between experts to facilitate shared learning, engagement, and research development. Public and patient involvement is key to creating meaningful research that addresses the real‐life difficulties for those living with, caring for someone with, or working with someone with a condition, and a growing body of evidence focuses solely on PPI or reports PPI involvement within its research.^1^, ^5^, ^20^, ^21^ However, the Liverpool Dementia & Ageing Research Forum appears to be the first CoP into dementia and ageing which advances individual types of public involvement and engagement, such as involving stakeholders in grant developments or dissemination, to forming a coherent Forum. This approach not only equitably involves people with personal and professional experiences in research, but also offers a platform to connect and share learnings with one another. Based on Wenger's^12^ original theory of Communities of Practice, this evidenced approach further adds to two reported monostakeholder‐specific dementia Communities of Practice^15^, ^16^ by reporting the benefits of multistakeholder involvement.

This increased involvement in research as one benefit of the Forum has led to improved capacity building, as evidenced in the reflections. One healthcare professional, working as an assistant psychologist supporting people with dementia and carers, has been actively involved in journal clubs, webinars, and other events and networking opportunities, which has raised his research capacity. Similarly, one unpaid carer has been strongly involved in various regular activities, whilst also being involved in different grant applications and funded research projects, all as a result of the Forum. While these are reflections, they illustrate how members of the Forum, from different backgrounds, can benefit from a topic‐specific CoP that enables equitable involvement. With a dearth of evidence on how Communities of Practice can raise research capacity, there is some evidence reporting on the impact of meaningful public involvement of people with dementia and improved skillsets as a result. Beresford‐Dent et al.^8^ reported improved skills in people with dementia and unpaid carers through their strategic involvement as public advisers in a randomised controlled trial. The authors also highlight though that improving research capacity required significant time and effort from the academic research team members. Further research needs to be conducted on the impact of PPIE activities and overarching or linked Communities of Practice on capacity building.

In addition to increased research capacity, responders also noted the impacts of the Forum on improving wider knowledge about dementia and latest research, with some respondents reporting improved access to support services as a result. Communities of Practice are known to enable knowledge exchange among members.^11^, ^16^ With a dearth of evidence into Communities of Practice in dementia and ageing, specifically in diverse stakeholder communities, this evaluation appears to be the first to showcase the positive impacts of the Forum on knowledge exchange. This is particularly important given the mix of members and attendees, as people with lived experiences of dementia are generally little able to easily connect with care providers and ask questions and discuss issues. The improved connectivity between different stakeholders, as well as the public talks about different services and research evidence, appear to have led some respondents to experience improved access to support services. Whilst only 11% (*n* = 5) of respondents reported this, from the pool of possible respondents to have experienced this outcome (*n* = 13 lived experts), this is an important finding. Considering the myriad of inequalities that most people with dementia and carers face when trying to access care,^22^, ^23^, ^24^ this evaluation provides the first and exploratory evidence as to how a public‐facing CoP into dementia, which offers a durable network built on quality relationships and trust and enables sharing of knowledge and awareness, has enhanced the skills and expertise of stakeholders, leading to changes in how people (i.e., carers and people with dementia) are able to access care.

## 5 CONCLUSIONS

The Liverpool Dementia & Ageing Research Forum is the first reported dementia‐specific CoP bringing together diverse stakeholders in the field. Setting up a dementia‐specific CoP can help raise knowledge and education, as well as awareness about the condition and care pathways, as well as enable research capacity, meaningful co‐produced research and improved access to care. While the Liverpool Dementia & Ageing Research Forum focuses on dementia, such a dementia‐specific CoP can be adapted to encompass or refocus on more medical aspects surrounding dementia. A future in‐depth evaluation is required to explore the experiences of engaging with the Forum and its impacts via qualitative interviews or focus groups with a larger group of attendees. This methods paper provides an approach to setting up and running dementia Communities of Practice elsewhere, and what to consider when doing so, representing a non‐interventional avenue to support those with lived and professional experiences in the field.

## AUTHOR CONTRIBUTIONS

Clarissa Giebel designed and set up the Liverpool Dementia & Ageing Research Forum, and designed the evaluation. Clarissa Giebel drafted the manuscript and analysed the survey responses. Ruth Eley, Hilary Tetlow and Thomas Faulkner wrote their sections in the Methods section, and jointly interpreted the findings and made recommendations with Clarissa Giebel. All authors approved the final manuscript.

## CONFLICT OF INTEREST STATEMENT

Clarissa Giebel is an Associate Editor of the journal and was not involved in any decision‐making surrounding this manuscript assessment. The remaining authors declare no conflict of interest.

## Data Availability

The data that support the findings of this study are available from the corresponding author upon reasonable request.

## Appendix A

**Table A1:** Evaluation survey.

#	Question	Possible answers
1	What is your background?	Academic Healthcare professional Social care professional Charity Local council/policy representative Student Lived expert (person with dementia/current or former carer) General public
2	Where are you from/do you work?	Liverpool region United Kingdom International
3	What type of activities have you engaged with or are engaging with on a regular basis?	Bimonthly public seminars/webinars Monthly journal clubs Regional networking meetings Annual conference
4	Approximately how many events have you participated into date?	1–5 5–10 10–20 20+
5	The aims of the Forum are twofold: (1) to raise awareness and inform and discuss the latest research and evidence in dementia and ageing; and (2) to connect professionals, students, volunteers and lived experts in the field to jointly generate ideas for research and improve dementia care. Do you feel these aims are met with the activities provided?	Yes No
6	Why do you attend the Forum events? Please tick all that apply	To find out more about the latest research and evidence To meet peers and connect with different stakeholders and experts in the field
7	Overall, what are your experiences of the Forum?	Very negative Negative Neutral Positive Very positive
8	Can you write a brief summary of your experiences and what the Forum has offered to you?	
